# A Case of Severe Hypoxia Caused by Phenazopyridine-Induced Methemoglobinemia: A near Fatal Event from Over-the-Counter Medication Use

**DOI:** 10.3390/clinpract12060089

**Published:** 2022-10-27

**Authors:** Ojbindra KC, Ananta Subedi, Rakshya Sharma, Punya Hari Dahal, Manisha Koirala

**Affiliations:** 1 Department of Hospital Medicine, Faith Regional Health Services, Norfolk, NE 68701, USA; 2 Department of Hospital Medicine, Avera McKennan Hospital and University Health Center, Sioux Falls, SD 57105, USA

**Keywords:** methemoglobinemia, hypoxia, phenazopyridine, over-the-counter medication

## Abstract

Methemoglobinemia is a rare blood disorder characterized by the oxidation of heme iron from ferrous (Fe^2+^) to ferric (Fe^3+^) state, which increases oxygen affinity and impairs oxygen release to the tissue causing hypoxia. It can be congenital or acquired; however, most cases are acquired and caused by exogenous substances such as medications, chemicals, and environmental substances. Phenazopyridine is an over-the-counter urinary analgesic medication commonly used for symptomatic relief of dysuria and has been reported to cause methemoglobinemia. However, only a handful of cases of phenazopyridine-induced methemoglobinemia have been reported. We present a case of an 89-year-old female who presented with severe hypoxia, shortness of breath, headache, nausea, and dizziness caused by phenazopyridine-induced methemoglobinemia. She was found to have a methemoglobin level of 21.5% and was treated with methylene blue, leading to a rapid improvement of her symptoms. She was taking one over-the-counter phenazopyridine 200 mg tablet three times daily for two weeks for her chronic dysuria. This case highlights the need to have a high index of suspicion of phenazopyridine-induced methemoglobinemia in a patient presenting with unexplained shortness of breath with a history of phenazopyridine use as it could lead to severe methemoglobinemia with hypoxia that could potentially be fatal if not promptly diagnosed.

## 1. Introduction

Methemoglobinemia is a rare blood disorder characterized by a higher-than-normal amount of methemoglobin in the blood [1]. Methemoglobin is an oxidized form of hemoglobin where heme iron is oxidized from ferrous (Fe^2+^) to ferric (Fe^3+^) state, which increases oxygen affinity and impairs oxygen release to the tissues, leading to hypoxia [1,2]. Methemoglobinemia is a rare cause of hypoxia and can be congenital or acquired [3]. However, most cases are acquired, induced by exogenous substances such as medications, chemicals, and environmental substances [4]. Phenazopyridine is a urinary analgesic medication used for symptomatic relief of dysuria and is available as an over-the-counter-medication. Phenazopyridine is known to cause methemoglobinemia; however only a handful of cases have been reported [5]. We report the case of an 89-year-old female who presented with severe hypoxia and shortness of breath caused by phenazopyridine-induced methemoglobinemia.

## 2. Case Report

An 89-year-old white female with a past medical history of hypothyroidism, hyperlipidemia, hypertension, chronic kidney disease stage III, and a history of recurrent urinary tract infection presented to the hospital with progressive shortness of breath for one-week duration. She reported shortness of breath at rest, which worsened with activity, along with headache, nausea, and dizziness. She denied chest pain, cough, fever, chills, palpitations, leg swellings, abdominal pain, and vomiting. She reported as having recovered from a COVID-19 infection three months ago and had been without any residual symptoms ever since. She had no previous history of similar presentation, and the review of drug allergies was unremarkable except for an allergic rash with nitrofurantoin. Her primary care physician evaluated her two days previously for shortness of breath. She was found to have a normal chest X-ray and normal blood work, including a complete blood count and comprehensive metabolic panel. However, her shortness of breath continued to worsen at rest and during activity, with a recorded saturation of 85% on room air at home, which made her present to the hospital.

In the emergency department, her initial vitals were a blood pressure of 127/73, a temperature of 36.7 °C, a heart rate of 60 beats per min, respiratory rate of 26 per min with a saturation of 82% on room air. She was placed on oxygen via a nasal cannula; however, oxygen saturation remained at 82–85% on 6 L oxygen via a nasal cannula. Subsequently, she was placed on bilevel positive airway pressure (BiPAP) with a fraction of inspired oxygen (FiO2) of 70% and oxygen flow rate of 14 L/min with an improvement of oxygen saturation to 90%. The physical examination revealed mild tachypnea and cyanosis of fingers and lips. However, the lung examination was unremarkable, with bilaterally equal air entry with no crackles or wheezing. The rest of the physical examination was unremarkable. 

The arterial blood gas (ABG), which was done on BiPAP with FiO2 of 70% and flow rate of 15 L/min, revealed an extremely high partial pressure of oxygen (PaO2) of 336 mmHg with normal pH, a partial pressure of carbon dioxide (PaCO2), and the concentration of bicarbonate (HCO3) which is highlighted in Table 1. The rest of the laboratory workups were unremarkable except for mild anemia, which is summarized in (Table 1). 

The respiratory viral panel and COVID-19 PCR were unremarkable. The chest X-ray was unremarkable, and CT chest angiography revealed mild bibasilar atelectasis with no evidence of pulmonary embolism. The echocardiography was unremarkable with no shunts. Despite using BiPAP, her oxygen saturation (SpO2) was 89–90%, and ABG revealed very high PaO2, suggestive of refractory hypoxia; additional workups of carboxyhemoglobin and methemoglobin were conducted, which revealed normal carboxyhemoglobin but an elevated methemoglobin of 21.5. Hence, a diagnosis of severe hypoxia due to methemoglobinemia was made. 

**Figure 1:**
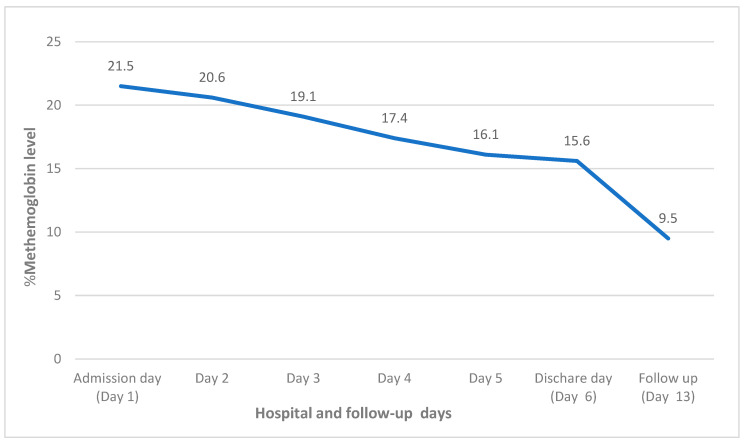
The trend of methemoglobin levels during the hospitalization and follow-up.

**Figure 2:**
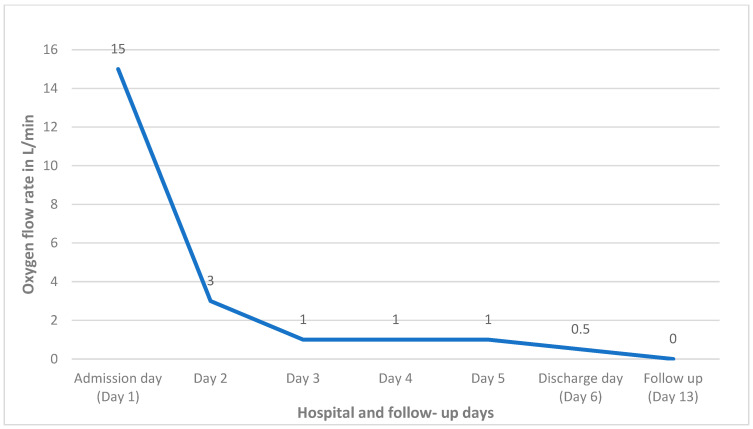
The trend of oxygen requirement during the hospitalization and follow-up.

On review of her home medications, phenazopyridine (Pyridium) was noted, which was prescribed as one tablet (200 mg) by mouth three times daily as needed for urinary discomfort. On further inquiry, she mentioned that she has been taking one tablet (200 mg) three times daily regularly for two weeks for urinary discomfort. She reported refilling the medication from over-the-counter without the physician’s prescription. She mentioned that phenazopyridine helped to subside the urinary discomfort; however, the dysuria had not resolved entirely. Her urine was analyzed, which revealed pus cells (WBC 10–20/HPF), and the culture grew Pseudomonas aeruginosa and Enterococcus faecalis. The urinary tract infection was treated with piperacillin-tazobactam antibiotics. For the methemoglobinemia-induced hypoxia, she was treated with methylene blue 1 mg/kg dose; two doses were administered on the first and second day of the hospitalization, respectively. She showed significant improvement in the hypoxia symptoms (Figure 1) with decreasing methemoglobin levels in the blood (Figure 2). She was discharged to the skilled nursing facility on the sixth day of hospitalization on 0.5 L/min continuous oxygen via nasal cannula. Her hypoxia and shortness of breath was found to be entirely resolved at the follow-up in her primary care physician’s office after one week of discharge. 

## 3. Discussion

Methemoglobinemia is characterized by the oxidation of heme iron from ferrous (Fe^2+^) to ferric (Fe^3+^) state, which increases oxygen affinity and impairs oxygen release to the tissue causing hypoxia [1,2]. Methemoglobin formation and conversion back to a normal ferrous state occur at a low level during normal red blood cell metabolism. At a steady state, the methemoglobin level is approximately 1% of total hemoglobin [1,2]. RBC enzyme cytochrome b5 reductase (Cyb5R) plays a major role in the conversion of methemoglobin to normal hemoglobin, and congenital deficiency of Cyb5R leads to congenital methemoglobinemia, which is primarily asymptomatic [6]. Acquired methemoglobinemia is more common than congenital methemoglobinemia, and is caused by various oxidizing substances such as medications (dapsone, chloroquine, primaquine, nitroglycerin, rasburicase, topical anesthetics such as benzocaine and lidocaine, sulfonamides, phenazopyridine) and chemical and environmental substances (anilines dyes, antifreeze, benzene derivatives, nitrates, and nitrites, hydrogen peroxide, paraquat, chorates, naphthalenes) [4]. The severity of symptoms from methemoglobinemia correlates with the methemoglobin level, which is summarized in (Table 2).

Phenazopyridine is an azo dye used as a urinary analgesic medication for symptomatic relief of dysuria and is available as an over-the-counter medication. It is a well-tolerated medication; however, it should not be used for more than two days due to potential adverse effects such as cytopenia, hemolytic anemia, nephrotoxicity, transaminitis, and methemoglobinemia [8]. Phenazopyridine is a known cause of acquired methemoglobinemia. However, only a handful of cases have been reported in the literature in the last twenty years, summarized in (Table 3) [5,8,9,10,11,12,13,14,15]. The various patient factors such as chronic kidney disease, heart and lung disease, anemia, and inherent enzymes such as G6PD deficiency, Cytochrome b5 reductase (Cyb5R) deficiency, or heterozygotes variant with a lower than average baseline activity increase susceptibility for phenazopyridine-induced methemoglobinemia [16]. Hence, methemoglobinemia could occur with variable amounts and duration of intake of phenazopyridine, as demonstrated in (Table 3). The use of Phenazopyridine in a patient with chronic kidney disease with creatinine clearance (CrCl) < 50 mL/min has been associated with acute kidney injury, hemolytic anemia, and methemoglobinemia; hence the use of Phenazopyridine should be avoided in such patients [17]. 

Methemoglobinemia should be suspected in patients with unexplained cyanosis and hypoxia that does not resolve with supplemental oxygen. The partial pressure of oxygen (PaO2) in ABG will be extremely high as a patient will be treated with a high level of supplemental oxygen; despite high PaO2, the oxygen saturation in pulse oximetry typically will be 80–90% which reflects the PaO2-saturation gap that would be indicative of methemoglobinemia [18]. Standard pulse oximetry cannot detect methemoglobin; Co-oximetry can detect methemoglobin at 630 nm, however it may be falsely positive in the presence of sulfhemoglobin [19]. Direct blood assay (Evelyn-Malloy Method) can quantify methemoglobin level in blood, which is useful when re-measurement of methemoglobin level is needed after treatment [19]. The treatment of acquired methemoglobinemia is to first identify the exposure, stop it, give supplemental oxygen, and provide supportive care. In symptomatic cases, where methemoglobin levels are usually >30%, 1–2 mg/kg of methylene blue, taken intravenously, can show rapid improvement; however, it should be avoided in patients with glucose-6 phosphate dehydrogenase deficiency(G6PD) as it may precipitate hemolysis, and should also be avoided in patients taking serotonergic medication due to the risk of serotonin syndrome [19]. In our patient, G6PD was not checked before administration of methylene blue as she was critically ill, and the test result would have taken 2–3 days. However, the patient was closely monitored for hemolysis and responded well to methylene blue with no evidence of hemolysis, which suggested she did not have G6PD deficiency. Ascorbic acid (Vitamin C) taken intravenously can be used in a patient where methylene blue is contraindicated [18]. Exchange transfusion and hyperbaric oxygen have been used in severe refractory cases of methemoglobinemia [20,21].

Our patient was an elderly lady who was prescribed phenazopyridine 200 mg tablet three times daily as needed for urinary discomfort; however, she was taking Phenazopyridine 200 mg three times daily for two weeks over-the-counter without the physician’s prescription. As a result, she developed shortness of breath and visited her primary care physician (PCP). She was not hypoxic at the PCP office, and her workup was unremarkable. However, her shortness of breath worsened with the development of severe hypoxia after two days of a PCP visit at the nursing home. This highlights that diagnosis of methemoglobin-induced hypoxia can be challenging. Therefore, a high index of suspicion and detailed knowledge of medical history, together with a thorough evaluation of prescription and over-the-counter medication use is needed. Our patient had chronic kidney disease stage III with a CrCl of 44 mL/min, which put her at a higher risk of developing methemoglobinemia from Phenazopyridine. Hence, physicians should take utmost care when prescribing Phenazopyridine in patients with reduced renal function, and patients should be strongly advised not to take the medication for more than two days. Our patient had a methemoglobin level of 21.5% but had severe symptomatic hypoxia as she had pre-existing chronic anemia. The Methylene blue was administered due to a high oxygen requirement with severe symptoms of dyspnea, nausea, headache, and dizziness. Rapid clinical improvement with resolution of symptoms occurred. 

## 4. Conclusions

Phenazopyridine is a commonly used over-the-counter medication for urinary discomfort but could lead to severe methemoglobinemia with hypoxia that could potentially be fatal if not promptly diagnosed. Therefore, this case highlights the need to strongly advise patients not to take phenazopyridine for more than two days and to have a high index of suspicion of phenazopyridine-induced methemoglobinemia in a patient presenting with unexplained shortness of breath with a history of phenazopyridine use.

## Figures and Tables

**Table 1:** The laboratory workup at the day of admission.

Variables	References	Observed Value
pH, arterial blood	7.35–7.45 units	7.39 units
pCO2, arterial	32–45 mmHg	43 mmHg
pO2, arterial	70–100 mmHg	336 mmHg
HCO3, arterial	21–28 mM/L	26 mM/L
Hemoglobin	12.0–15.5 gm/dL	10.3 gm/dL
White blood cell count	4.0–10.0 × 10^3^/µL	7.8 × 10^3^/µL
Platelets	150–450 × 10^3^/µL	232 × 10^3^/µL
Sodium	135–146 mEq/L	138 mEq/L
Potassium	3.5–5.3 mEq/L	4.9 mEq/L
Chloride	98–110 mEq/L	107 mEq/L
Bicarbonate	21–30 mM/L	24 mM/L
Blood urea nitrogen	7.0–30 mg/dL	54 mg/dL
Creatinine	0.55–1.02 mg/dL	1.35 mg/dL
Glucose	65–99 mg/dL	99 mg/dL
Glomerular filtration rate (GFR)	CKD if < 60 mL/min/1.73 sq m	35 mL/min/1.73 sq
D-dimer	<230 ng/mL	<150 ng/mL
Chronic heart failure peptide (BNP)	0–100.0 pg/mL	30.0 pg/mL
Procalcitonin	<0.10 ng/mL	0.14 ng/mL
Methemoglobin	0–1.5%	21.5%
Carboxyhemoglobin	<1.5% non-smoker <5.0% smoker	0.0

**Table 2:** Severity of symptoms of methemoglobinemia with methemoglobin level [7].

Methemoglobin Level	Symptoms
<0–3% (mean: 1%)	Normal range for adults
3–12%	Minimal level, cyanosis may present at level above 5–10%
13–20%	Usually asymptomatic unless preexisting condition such as anemia, heart and lung disease are present that may exacerbate toxicity
20–50%	Mild to moderate symptoms such as lightheadedness, fatigue, tachycardia, dyspnea, and lethargy
50–70%	Severe symptoms such as altered sensorium, respiratory depression, shock, seizures and coma
>70%	Usually fatal

**Table 3:** The previous cases of phenazopyridine-induced methemoglobinemia reported in literature.

Author	Age/Sex	Comorbidities	Symptoms	Dose of Phenzopyridine	Methemoglobin Levels	Treatment
Shahani L. et al. [5]	74/F	Chronic obstructive pulmonary disease, hypertension	Shortness of breath	Phenazopyridine for a month	18.9%	Methylene blue
Singh N.K. et al. [8]	79/F	Recurrent urinary tract infection	Purple hands, hypoxia	200 mg 3 times daily for 10 days	11.8%	Methylene blue
Agrawal A. et al. [9]	55/F	Multiple sclerosis, neurogenic bladder	Lethargy, suprapubic pain and hypoxia	200 mg TIDS for 5 months	9.3%	Pyridium discontinued
Yu C.H. et al. [10]	78/F	CKD stage III	Purple lips and hands, hypoxia	Phenazopyridine for 5 days	37.4%	Methylene blue
Murphy T. et al. [11]	57/F	Breast cancer, seizures, recent dysuria	Lethargy, light-headedness, headache, hypoxia.	24 tablets in 48 h	24.2%	Methylene blue
Hamza A. et al. [12]	55/F	Urinary retention with chronic foley catheter, pulmonary embolism	Dusky skin and hypoxia	400 mg TID for 2 months	22%	Vitamin C used as methylene blue was contraindicated due to use of mirtazapine
Banimah F. et al. [13]	32/F	No comorbidities	Lower abdominal pain, dysuria	1 tablet 3 times daily for 4 days	11.9%	Methylene blue
Shah P.K. et al. [14]	17/M	Obesity, cerebral palsy, muscular dystrophy	Desaturation with higher oxygen requirement	200 mg three times daily for 2 days	15.8%	Phenazopyridine discontinued
Shatia W. et al. [15]	28/F	Chronic interstitial cystitis	Acute on chronic suprapubic pain, hypoxia	200 mg 3–4 tablets daily	22.2%	Methylene blue, riboflavin and ascorbic acid.
